# SINO Syndrome Causative KIDINS220/ARMS Gene Regulates Adipocyte Differentiation

**DOI:** 10.3389/fcell.2021.619475

**Published:** 2021-03-04

**Authors:** Kaihui Zhang, Wenxing Sun, Yi Liu, Yuqiang Lv, Daisen Hou, Yan Lin, Wei Xu, Jianyuan Zhao, Zhongtao Gai, Shimin Zhao, Yiyuan Yuan

**Affiliations:** ^1^The Obstetrics and Gynecology Hospital, Fudan University, Shanghai, China; ^2^Pediatric Research Institute, Qilu Children’s Hospital of Shandong University, Ji’nan, China; ^3^State Key Lab of Genetic Engineering and School of Life Sciences, Fudan University, Shanghai, China; ^4^Department of Nutrition and Food Hygiene, School of Public Health, Nantong University, Nantong, China; ^5^Key Laboratory of Reproduction Regulation of NPFPC, Institutes of Biomedical Sciences and Collaborative Innovation Center of Genetics and Development, Fudan University, Shanghai, China

**Keywords:** SINO syndrome, KIDINS220/ARMS, adipocyte differentiation, intellectual disability, obesity, brachycephaly

## Abstract

Nonsense variants in KIDINS220/ARMS were identified as the main cause of spastic paraplegia, intellectual disability, nystagmus, and obesity (SINO) syndrome, a rare disease with birth defects in brachycephaly, neurological disorder, and obesity. The cause of neural cell dysfunction by KIDINS220/ARMS were extensively studied while the cause of obesity in SINO syndrome remains elusive. Here, we identified KIDINS220/ARMS as an adipocyte differentiation-regulating gene. A Chinese family, mother and her two sons, all showed severe symptoms of SINO syndrome. G-banding karyotyping, chromosome microarray analysis, and whole exome sequencing revealed a novel amber mutation, c.3934G>T (p. E1312X), which was close to the C-terminal region of KIDINS220/ARMS and resulted in the premature of the protein. Both the mRNA and protein levels of KIDINS220/ARMS gradually decreased during adipocyte differentiation. Knockdown of KINDINS220/ARMS could prompt adipocyte differentiation and lipid accumulation while overexpression of KIDINS220/ARMS decrease the rate of matured adipocytes. Furthermore, we demonstrated that KIDINS220/ARMS inhibits adipocyte maturation through sustained extracellular signal-regulated kinase signaling. In conclusion, this is the first report about a vertical heredity of severe dominant pathogenic mutation of KIDINS220/ARMS, suggested that KIDINS220/ARMS played a negative role in adipocyte maturation, explained the cause of obesity in SINO syndrome and could highlight the importance of adipocyte differentiation in neuron functions.

## Introduction

The syndrome of spastic paraplegia, intellectual disability, nystagmus, and obesity (SINO; MIM# 617296) is an autosomal dominant rare disease with birth defects of cranial and maxillofacial deformity, severe intellectual disability, and obesity, which was first described and linked to heterozygous nonsense kinase D-interacting substrate of 220 kDa (*KIDINS220/ARMS*) mutations by Josifova et al. (2016) in three unrelated patients. *KIDINS220/ARMS*, also known as ankyrin repeat-rich membrane spanning (*ARMS*), encodes a conserved membrane protein that is mainly expressed in the nervous system (Iglesias et al., 2000; Bracale et al., 2007). The putative KIDINS220/ARMS structure in humans contains 11 ankyrin repeats, four transmembrane domains in a KAP region, a proline-rich domain (PRD), a sterile alpha motif (SAM), kinesin light chain (KLC)-interactive motif (KIM), and a PDZ ligand (PDZ-L) from the N-terminal (Nt) to the C-terminal (Ct), which are both exposed to the cytoplasm (Iglesias et al., 2000; Kong et al., 2001). As a transmembrane protein, KIDINS220/ARMS functions as a scaffold to organize the signaling complex and transduce the extracellular stimuli to cellular response and plays crucial roles in diverse cellular process, such as neuronal cell survival and synaptic plasticity in the nervous system (Zampieri and Chao, 2006; Jaudon et al., 2020), vascular and heart development (Cesca et al., 2012; Mero et al., 2017), as well as B cell and T cell development and activation in the immune system (Deswal et al., 2013; Fiala et al., 2015). In addition to its functions in cellular biology and development, growing evidences have also linked KIDINS220/ARMS to different pathologies of human diseases, including Alzheimer’s disease (AD), asthma, and cancer (Ni et al., 2010; Lopez-Menendez et al., 2013; Raza et al., 2018).

Due to its pivotal roles in development and diverse cellular process, Kidins220/ARMS knockout mice are embryonically lethal at the late stage of gestation with developmental defects in the nervous and cardiovascular system (Cesca et al., 2012). In humans, homozygous truncated variant in KIDINS220/ARMS caused enlarged cerebral ventricles and limb contractures of fetuses and thus pregnancy terminations (Mero et al., 2017). It seemed that heterozygous mutations were tolerated, since the heterozygous mouse strain was successfully generated at New York University, although the mice had reduced dendritic complexity and spine instability (Duffy et al., 2011). In humans, children carrying the heterozygous mutations of KIDINS220/ARMS showed SINO, which was first identified in three unrelated children and was named “SINO syndrome” by Josifova et al. (2016). Currently, six heterozygous causative mutations in *KIDINS220/ARMS* had been identified as the causes of SINO syndrome in children (Josifova et al., 2016; Yang et al., 2018; Zhao et al., 2019). Patients with SINO syndrome usually had delayed neurological development due to the fundamental functions of KIDINS220/ARMS in the nervous system (Josifova et al., 2016). Interestingly, obesity was also found in almost all SINO patients; however, it is not clear whether obesity of SINO patients is caused by KIDINS220/ARMS dysfunction or due to impaired movement ability. Therefore, the potential role of KIDINS220/ARMS in fat metabolism or adipocytes regulation was largely ignored.

In this study, we identified a novel truncated mutation of KIDINS220/ARMS in a familial SINO syndrome. To elucidate the possible functions of KIDINS220/ARMS in adipose cell that might cause obesity, we studied the mechanisms of KIDINS220/ARMS in the adipogenic differentiation process. Our results suggested that KIDINS220/ARMS, through sustaining the phosphorylation of extracellular signal-regulated kinase (ERK), could function as a negative regulator for the adipocyte differentiation, thus the truncated mutation of KIDINS220/ARMS could lead to uncontrolled differentiation and maturation of adipose cells, which caused obesity in SINO patients.

## Materials and Methods

The study was carried out in compliance with the Helsinki Declaration and approved by the hospital ethics committees of Qilu Children’s Hospital of Shandong University (ETYY-2017012). The husband/father of the patients gave their written informed consents before clinical and laboratory examinations. The information of the patients was anonymized prior to submission.

### G-Banding Karyotyping and Chromosome Microarray Analysis

Peripheral blood leukocytes from the mother and her children were stimulated by phytohemagglutinin. Metaphase chromosome G-banding karyotyping was performed at the 400 bands of resolution following standard procedures. The analysis was carried out using imaging software CytoVision (Leica, Frankfurt, Germany) according to the International System for Human Cytogenetic Nomenclature (ISCN) (Simons et al., 2013).

Illumina SNP array (InfiniumOmniZhongHua-8, 900K, Illumina, San Diego, CA, United States) was used to detect the copy number variations (CNVs), insertions-deletions (INDELs), single nucleotide polymorphisms (SNPs), and structural variants in the whole genome of the younger boy. The standard operation procedure was performed according to the Illumina protocol. The obtained data were analyzed using Illumina’s KaryoStudio software and the cnvPartition algorithm for chromosome information detection and then combined with the Database of Genomic Variant (DGV) database for sequential screening to effectively detect the CNV region. For result annotation, a combination of the software’s own database and other databases, such as Decipher, Clinvar, UCSC, ISCA, etc., were used.

### Whole Exome Sequencing and Bioinformatics Analysis

Whole exome sequencing (WES) was performed for the gene mutation screening of the younger boy. DNA library was prepared and enriched according to the manufacturer’s protocol (SureSelectXT Automated Target Enrichment for Illumina Pair-End Multiplexed Sequencing; Agilent Technologies, Santa Clara, CA, United States). The enriched libraries were sequenced using the HiSeq 2000 sequencing system (Illumina, San Diego, CA, United States). Bioinformatics analysis and annotation were carried out by NextGene V2.3.4 software (SoftGenetics, State College, PA, United States) and self-developed scripts using 1000 Genomes, ExAC, gnomAD, dbSNP, HGMD, Clinvar, and OMIM databases as reference database to generate annotation information, such as conservation of bases and amino acids, prediction of biological function, frequency of normal population, etc. Genetic variants, such as small INDEL, typical splicing site changes and missense variants, the suballelic frequency in the normal population <5%, and genetic variants included in HGMD and ClinVar databases, were further analyzed. To verify the mutation, PCR and capillary Sanger sequencing were carried out for the parents and his brother and for the parents and one of the siblings of the mother. Some *in silico* databases were used to define the variants, such as UCSC, PolyPhen-2, and Mutation_Taster. The genotype-phenotype correlation was analyzed with a pediatrics and genetic counselor. The pathogenicity of genetic variation was evaluated according to the standards and guidelines for sequence variation published by the American Society of Medical Genetics and Genomics (ACMG) in 2015 (Richards et al., 2015), and HGV nomenclature was adopted.

### Cell Culture

The 3T3-L1 cells (ATCC CL-173^TM^) were cultured in Dulbecco’s modified Eagle’s medium (DMEM; Hyclone) containing 10% new-born calf serum (NCS, Gibco-Invitrogen, Carlsbad, CA, United States), and 100 IU/ml penicillin-streptomycin in a 5% CO_2_ incubator at 37°C. After 2 days of post-confluence, the 3T3-L1 cells were induced to differentiation by DMEM including 10% fetal bovine serum (FBS; Hyclone), 1 μg/ml insulin, 1 μM dexamethasone, and 0.5 mM 3-isobutyl-1-methylxanthinem for 2 days. Then, the medium was switched to DMEM with 10% FBS and 1 μg/ml insulin for 2 days, followed with DMEM containing 10% FBS every other day.

### Cell Transfection and RNA Interference

Human *KIDINS220/ARMS* and Truncated *KIDINS220/ARMS* (p.E1312^∗^) were cloned into pcDNA3.1(b+)-C-Flag. Cells were harvested 36 h later after transfection. In the 3T3-L1 cells, the knockdown of *KIDINS220/ARMS* gene was carried out using synthetic siRNA oligonucleotides (Genepharma) with Lipo8000^TM^ (Beyotime). Preadipocytes were transiently transfected by electroporation using Amaxa Nucleofector II Electroporation Machine (Lonza). Briefly, 1 × 10^6^ cells were suspended in 100 ml DMEM, mixed with 2 mg plasmid, and then transfected by a pulse of electroporation using T-030 program. The sequences for primers and siRNA are listed in Supplementary Table 1. For preadipocyte induction, KIDINS220-Flag or KIDINS220 siRNA were transfected to 3T3-L1 cell and cultured for 2 days. The 3T3-L1 cells were induced to differentiation (Day 0) and transfected with KIDINS220 or KIDINS220 siRNA again on Day 1 using A-033 program. In addition, 1 mM Ravoxertinib or 10 mM Honokiol (Selleckchem Inc.) were added to the medium respectively on Day 1.

### Quantitative RT-PCR

Total RNA was extracted using Trizol reagent (Invitrogen), and the first-strand cDNA was synthesized from 1 μg total RNA with HiScript III 1st-Strand cDNA Synthesis Kit (Vazyme). Quantitative PCR was performed using the ChamQ SYBR Color qPCR Master Mix (Vazyme) with the standard procedure. Primer sequences used in this study are shown in Supplementary Table 1, and *Rplp0* was used as the endogenous control.

### Western Blotting

Cells were lysed using lysis buffer (0.05 M Tris–HCl, pH 6.8, 2% SDS, 1% b-mercaptoethanol, 10% glycerol, and 0.002% bromphenol blue). Protein concentrations were determined using the BCA Protein Assay Kit (Beyotime). Then, aliquots of 30 mg protein were used for western blotting. Proteins were separated by SDS-PAGE using a 10–250-kDa protein ladder (Precision Plus Protein^TM^-dual color, Bio-Rad), then transferred to nitrocellulose membrane (Millipore), and immunoblotted with antibodies (anti-Flag and anti-actin monoclonal antibody, Sigma; anti-KIDINS220 antibody from Abcam) according to standard procedures. Detection and quantification were done on a Typhoon FLA9500 (GE Healthcare) scanning system using an acridan-based chemiluminescent and chemifluorescent horseradish peroxidase (HRP) substrate (Pierce ECL Plus, Thermo Fisher Scientific).

### Oil Red O Staining

Levels of stored triglycerides in differentiated mature adipocytes were verified by staining intracellular lipid droplets with Oil red O (Solarbio) subjected to staining procedures as described previously (Oh et al., 2019). Images were taken using Olympus IX73 Inverted Microscope. To quantify the lipid content, Oil Red O was extracted with 100% isopropanol and calculated by the measuring absorbance at 490 nm using a SpectraMax i3x microplate reader (Molecular Devices, San Jose, CA, United States).

### Statistical Analysis

Data were analyzed using the GraphPad prism 8 software (GraphPad Software). All experimental data were performed at least three times, and values are presented as means ± standard error of measurement (SEM). Student’s unpaired *t*-test was applied to calculate the two-tailed *p*-value, and group differences were considered significant for ^∗^*p* < 0.05, ^∗∗^*p* < 0.01, ^∗∗∗^*p* < 0.001, and ^****^p < 0.0001.

## Results

### A Novel Pathogenic Mutation of KIDINS220/ARMS Causes SINO Syndrome and Obesity

#### Clinical Characteristics and Case Following-Up

**FIGURE 1:**
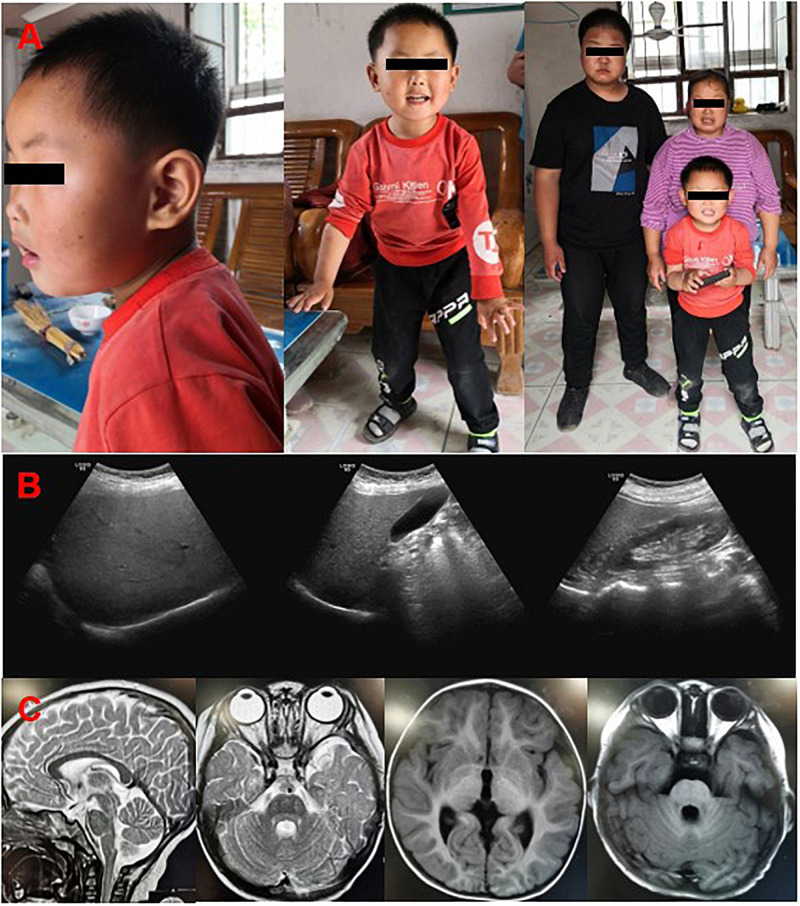
Clinical features of the patients. **(A)** The pictures of the probands, mother and her two sons in the family by following-up. **(B)** Color Doppler ultrasound of mother showed that liver section is normal in size, smooth in capsule, dense in liver parenchyma, uniform in distribution, and clear in tube structure. **(C)** Brain MRI of the younger son showed that no abnormal density lesions were found in the cerebral parenchyma, and cerebrospinal fluid cavity, the ventricle, sulcus, and cleft lobes were normal, the brain gyrus was clear, and the midline structure was centered.

A 36-year-old woman was highly suspected as PCOS with the clinical manifestations of irregular menstruation and obesity in the gynecological department. The patient showed obvious psychomotor retardation, and we noticed that both of her two sons had the same or even worse symptoms during the diagnosis process (Figure 1A). Thus, she was recommended to the neurologist and genetic counselor. Her parents and siblings had normal intellect, but she presented obvious intellectual and physical disability. On neurological examinations, she was diagnosed as spastic paraplegia with the characteristic of hypermyotonia in both lower extremities, hyperactivity of tendon reflex, and scissors gait. Intellectual disability (Wechsler Adult Intelligence Scale: IQ = 39), nystagmus, and severe obese (BMI = 35.6, weight = 80 kg, height = 150 cm) were also diagnosed. Abdominal ultrasound revealed mild fatty liver (Figure 1B), while the biochemical tests showed that fasting blood glucose (4.9 mM), blood lipids (cholesterol = 4.7 mM; HDL-c = 1.35 mM; LDL-c = 2.36 mM; triglyceride = 1.65 mM), and liver enzymes (ALT = 20 U/L; AST = 28 U/L) were all normal.

Her elder son, who was 14 years old, had the similar but more serious symptoms of intellectual disability, speech impairment, spastic paraplegia, nystagmus, brachycephaly, and obesity (BMI = 29.4, weight = 85 kg, height = 170 cm). The second son, who was 19-month old in his first check-in, had a thorough physical examination by pediatricians. The gestation period was normal, and he was delivered normally at 40 weeks of gestation. The Apgar score was 9 with the weight of 3.8 kg and 52 cm in length when he was born, which was normal in Chinese population. His physical development was rapid, and both his weight and height are above the 99th percentile in Chinese population (weight = 15.3 kg, height = 90 cm). He showed macrocephaly (head circumference = 47.5 cm), brachycephaly, and non-verbal although his audiology test indicated he had no problem in hearing. He could raise his head at 12 months and roll over at 18 months, which is about 9 months delay than other infants. He can walk only accompanied by other person with the scissors gait (Supplementary Video 1). His motor development demonstrated severe delay for gross motor quotient (<1 percentile), fine motor quotient (=1 percentile), and total motor quotient (<1 percentile) using the Peabody Developmental Motor Scale (PDMS). The Gesell Developmental Observation-Revised (GDO-R) test exhibited extremely severe developmental delay for adaptability (score: 22 points), severe delay for language (score: 42 points), and personal-social interaction (score: 32 points), all indicated severe intellectual disability. Although he had mental retardation, the brain MRI showed that there has no abnormal density lesion in the cerebral parenchyma and cerebrospinal fluid cavity. The ventricle, sulcus, and left lobes were all normal, with clear brain gyrus, and the midline structure (Figure 1C). Interestingly, nystagmus, a symptom of SINO syndrome which was also found in both mother and elder brother, was not noticed in the second son.

The family was followed up 3 years later (Figure 1A). The second son, who is age five now, improved greatly in his intellectual development. He can speak simple words (mainly two to three Chinese characters) and communicate with other persons. He can stand and walk without any help, although the scissors gait is obvious. The GDO-R test exhibited moderate developmental delay for adaptability (score: 45 points), severe delay for language (score: 47 points), and mild delay for personal-social interaction (score: 62 points), all indicated moderate mental retardation which is better than 3 years ago. Both the mother and her two sons still have obesity problem, despite their regular and normal amount of food intake based on several eating and behavior questionnaire (Supplementary Table 2; Rockett et al., 1997; Ainsworth et al., 2000; Caccialanza et al., 2004; Vasheghani-Farahani et al., 2011). They can take care of themselves, and the elder brother learned to read and write in a school now. However, he still has severe intellectual problem and his Wechsler Adult Intelligence Scale is 42, he could only calculate addition and subtraction within 10, and recognize no more than 100 words.

#### Molecular Findings

**FIGURE 2:**
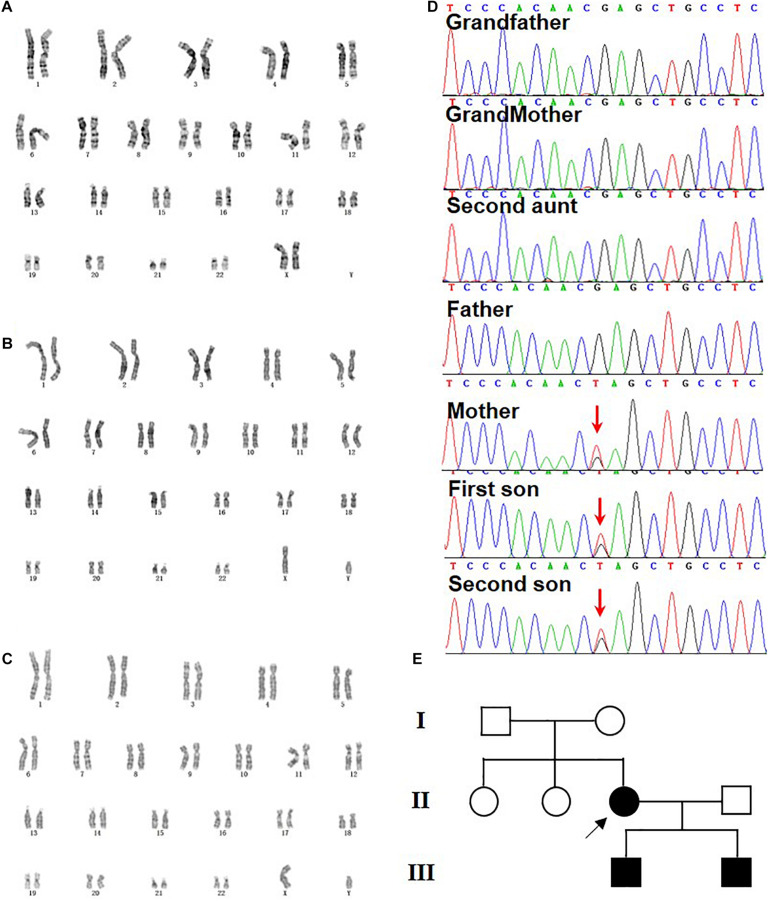
Genetic analysis and pedigree chart of the family. **(A–C)** G-banding karyotyping from mother (46, XX) **(A)**, elder son (46, XY) **(B)**, and second son (46, XY) **(C)**. **(D)** Sanger sequencing confirmation of heterozygous nonsense mutation c.3934G>T (p.E1312*) of KIDINS220/ARMS detected *via* WES in mother and her two sons. The arrow indicates the G to T mutations. The grandparents, the second aunt, and the father were normal. **(E)** The pedigree chart of the family and the arrow pointed the *de novo* mutation from the mother.

To demonstrate the heredity and genetic causes of the family case, G-banding karyotyping, chromosome microarray analysis, and WES were performed [full data sets could be found in Supplementary Material, and the amplicon sequencing reads for variant calling had been deposited with NODE Bioproject (OEP001230 and OEP001231)]. No chromosomal abnormalities were detected in this case (Figures 2A–C), but a novel heterozygous variant (c.3934G>T) in the exon 28 of *KIDINS220/ARMS* (NM_020738.2) was found and confirmed by Sanger sequencing in all three probands (Figure 2D). The original c.3934G nucleotide and the codon amino acid Glu1312 residue were highly conserved from xenopus to human (Supplementary Figure 1), suggesting a critical importance of this residue, while the c.3934G>T mutation resulting in GAG (Glu) leads to TAG (amber) at amino acid residue 1312. Meanwhile, the mutation was *de novo* to the mother since the Sanger sequencing of the *KIDINS220/ARMS* region revealed that her parents and one of the sisters were all normal (Figures 2D,E), and this novel variant was never reported in any main databases (Supplementary Figure 2 and Supplementary Table 3), such as the ExAC, the 1000 Genomes, and the gnomAD. The possible value of the mutation within *KIDINS220/ARMS* for the disease-causing variant was analyzed by Mutation_Taster software and can be classified as a pathogenic mutation according to the ACMG guidelines. This is the first time to report that a single site mutation in *KIDINS220/ARMS* could be transmitted in two generations, which leads to a severe autosomal dominant disease, SINO syndrome.

### KIDINS220/ARMS Overexpression Inhibits Adipocyte Hyperplasia

**FIGURE 3:**
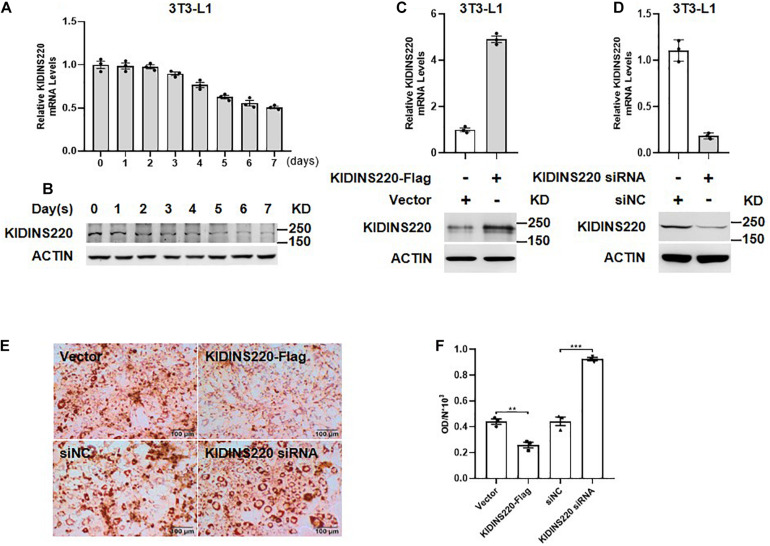
KIDINS220/ARMS inhibited adipocyte differentiation. The mRNA **(A)** and protein **(B)** levels of KIDINS220/ARMS are gradually decreased along with the differentiation process of preadipocyte 3T3-L1 cells. The overexpression **(C)** and knockdown **(D)** of KINDIS220/ARMS in 3T3-L1 cells were confirmed by quantitative PCR and immunoblotting. **(E)** 3T3-L1 preadipocytes were transfected with KIDINS220-Flag or KIDINS220 siRNA, respectively, and then induced to differentiation 48 h later (Day 0). On Day 8, cytoplasmic triacylglycerol was stained with Oil Red O. **(F)** The densities of staining were calculated by the absorbance at 490 nm. Student’s unpaired *t*-test; **p < 0.01; ***p < 0.001. siNC, scramble siRNA. Bar, 100 mm. All experimental data were performed at least three times.

The symptoms of spastic paraplegia, intellectual disability, and nystagmus could be explained by the key roles of KINDIS220/ARMS in neuronal cell survival, differentiation, and synaptic modulation, but how the truncated KIDINS220/ARMS causes severe obesity remains unclear. Since both the physical activity and food intake were normal based on our follow-up, the obesity may not be caused by the lower physical activity and higher calorific intake due to intellectual disability. Thus, KINDIS220/ARMS might have the potential roles in fat metabolism or adipocyte regulation. To address whether KIDINS220/ARMS had functions in adipocyte differentiation, which was critical to the onset of obesity in childhood (Hausman et al., 2001), we examined the expression level of KINDIS220/ARMS during adipocyte maturation. Both the mRNA and protein levels were gradually decreased according to the differentiation progress of 3T3-L1 cell (Figures 3A,B), which suggested KIDINS220/ARMS might play a negative role in adipocyte differentiation. We also noticed that when we overexpressed KIDINS220/ARMS in 3T3-L1 cells (Figure 3C), the lipid accumulation was significantly reduced, while knockdown of KIDINS220/ARMS by RNAi (Figure 3D) could prompt 3T3-L1 cell maturation and increases the storage of lipids (Figures 3E,F). Taken together, KIDINS220/ARMS could play a negative role in adipocyte differentiation, which was opposite to its positive functions in the nervous system. The truncated form of KIDINS220/ARMS found in SINO patients might lose its biological functions in controlling adipocyte differentiation, thus leading to uncontrolled adipocyte maturation, lipid accumulation, and obesity.

### ERK Pathway Is Required for KIDINS220/ARMS-Mediated Inhibition of Adipocyte Maturation

**FIGURE 4:**
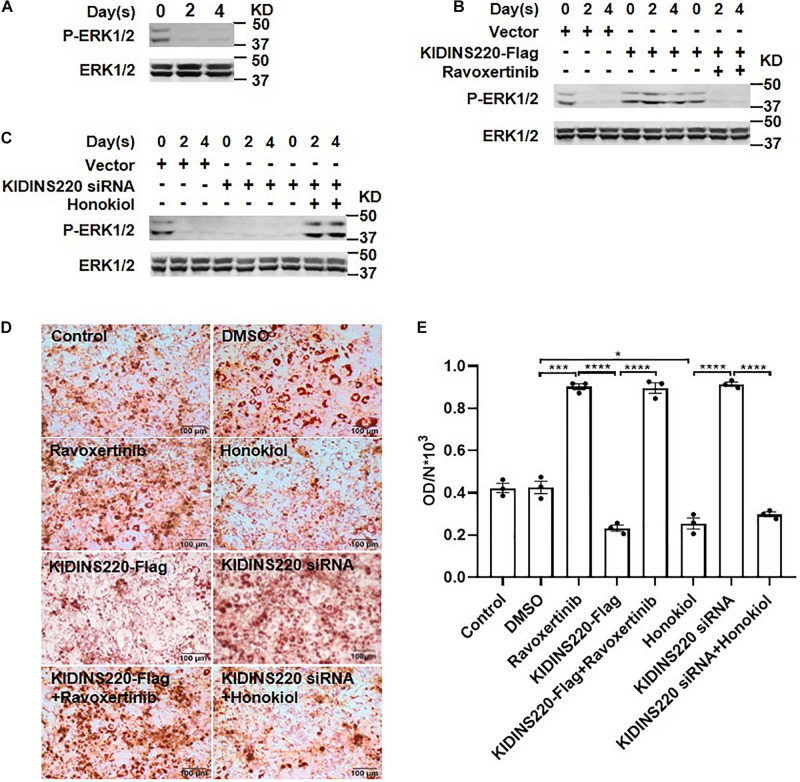
ERK pathway is required for KIDINS220/ARMS mediated adipocyte maturation. **(A)** 3T3-L1 cells were induced to differentiation, and cells were harvested for western blot analysis on Days 0, 2, and 4. **(B,C)** 3T3-L1 cells were transfected with KIDINS220-Flag **(B)** or KIDINS220 siRNA **(C)** and cultured for 2 days. Then, cells were induced to differentiation (Day 0), and ERK inhibitor (Ravoxertinib) **(B)** or agonist (Honokiol) **(C)** were added to the medium at Day 1, respectively. On Days 0, 2, and 4, cells were harvested and analyzed by western blot for the phosphorylation of ERK. **(D)** Images of Oil-red O staining performed 8 days after the differentiation. Ravoxertinib and Honokiol: cells treated with 1 mM Ravoxertinib or 10 mM Honokiol alone; KIDINS220-Flag and KIDINS220 siRNA: cells were transfected with KIDINS220-Flag or KIDINS220 siRNA, respectively; KIDINS220-Flag + Ravoxertinib: cells were transfected with KIDINS220-Flag and treated with 1 mM Ravoxertinib; KIDINS220 siRNA + Honokiol: cells were transfected with KIDINS220 siRNA and treated with 10 mM Honokiol. **(E)** The densities of staining were calculated by the absorbance at 490 nm. Student’s unpaired *t*-test; *p < 0.05; **p < 0.01; ***p < 0.001; ****p < 0.0001. Bar, 100 mm. All experimental data were performed at least three times.

It has been shown that ERK played a pivotal role in adipogenesis (Bost et al.,2005a,b). Thus, we investigated whether ERK acts as a downstream effector for KIDINS220/ARMS-mediated inhibition in adipocyte maturation. During the differentiation of preadipocytes, the ERK was dephosphorylated to prompt adipocyte maturation and lipid accumulation (Figure 4A). Overexpression of KIDINS220/ARMS constitutively activated the ERK signaling and inhibited adipocyte differentiation. Ravoxertinib (Sulahian et al., 2019), a highly selective ERK inhibitor which tuned down the ERK signaling (Figure 4B), could fully restore the ability of preadipocytes to differentiate to mature adipocytes and greatly increase the lipid storage (Figures 4D,E). In contrast, stimulating the ERK pathway by Honokiol (Lone and Yun, 2017) after induction (Figure 4C) could keep the preadipocyte in an undifferentiated status (Figures 4D,E). Therefore, our data support that KIDINS220/ARMS negatively regulates adipocyte maturation through ERK signaling pathway.

## Discussion

The development and differentiation of the nervous system during embryogenesis is highly orchestrated in which the neurotrophic factors play crucial roles to regulate the survival and differential of neurons (Lopez-Menendez et al., 2009). KIDINS220/ARMS, a scaffold protein which contains multiple functional domains (Supplementary Figure 2) that can transduce the neurotrophin (NT) signaling into cellular response, was cloned from neural cells and identified as the first substrate of protein kinase D (PKD) (Iglesias et al., 2000). KIDINS220/ARMS is generally expressed in all tissues, especially high in the nervous and cardiovascular system (Cesca et al., 2012; Mero et al., 2017). Homozygous mutations of KIDINS220/ARMS cause pregnancy terminations in humans (Mero et al., 2017), as well as the heterozygous truncation of KIDINS220/ARMS can lead to a neurological disorder named SINO syndrome (Josifova et al., 2016; Yang et al., 2018; Zhao et al., 2019).

Here, we identified a novel *KIDINS220/ARMS* amber mutation in *KIDINS220/ARMS* which caused SINO syndrome in a Chinese family. The mother and her two sons showed typical symptoms including brachycephaly, intellectual disability, and obesity. It is surprising that although the KIDINS220/ARMS has pivotal functions in development and is highly expressed in reproductive organs, such as the ovaries, the truncated protein does not affect female fertility. SINO patients usually have brachycephaly and enlarged head circumference. In this case, we also noticed that both sons showed macrocephaly, but how mutations of KIDINS220/ARMS mutation affect skull development remains unknown and could be studied later. Intellectual disability is a generalized neurodevelopmental disorder with features of significantly impaired intellectual and adaptive functioning. According to the Wechsler Adult Intelligence Scale, scores were lower for the mother and her two sons. The pivotal roles of KIDINS220/ARMS in the nervous system, including regulating the neural cell survival and differentiation, guiding the axon and dendrite growth, and modulating the synaptic transmission in motor neurons, could well explain the pathogenesis of intellectual disability, although the detailed mechanisms need to be further studied. However, why nonsense mutations of KIDINS220/ARMS lead to obesity remains unknown and warrants further investigation (Arevalo et al., 2006; Josifova et al., 2016; Kleinendorst et al., 2018).

People with intellectual disability have greater prevalence to develop obesity (Yamaki, 2005), possibly due to lower physical activity and higher calorific intake. Obesity is also a remarkable symptom of SINO patients, but whether it is caused by KIDINS220/ARMS dysfunction in fat metabolism and adipocytes regulation, or due to impaired movement ability and more food intake remains unclear. The previous reports (Josifova et al., 2016) and our case showed that obesity arose from infancy period or neonatal period without hyperphagia. We also observed that both the mother and her two sons have regular physical activity and normal amount of food intake, which indicated that KIDINS220/ARMS may have potential roles in fat metabolism and adipocyte regulation. Adipocyte hyperplasia, a result of preadipocyte differentiation, has been shown to play a critical role at the onset of obesity in all stages of life, particularly in childhood (Hausman et al., 2001). Here, to elucidate the possible functions of KIDINS220/ARMS in the pathogenesis of obesity, we examined the role of KIDINS220/ARMS in preadipocyte differentiation. Our results showed that during the process of adipocyte differentiation, both the mRNA and protein level were gradually downregulated in a time-dependent manner. Meanwhile, overexpression of KIDINS220/ARMS in 3T3-L1 inhibits its differentiation, while knockdown of KIDINS220/ARMS promotes adipocyte maturation and triacylglycerol accumulation. ERK is well known to play a pivotal role in adipogenesis (Bost et al.,2005a,b). Phosphorylation of ERK to initiate adipogenesis, as well as dephosphorylation of ERK after initiation is critical for adipocyte maturation since prolonged ERK phosphorylation inhibits adipogenesis (Ferguson et al., 2016). Accumulated evidences have indicated that KIDINS220/ARMS could phosphorylate and sustain ERK signaling in different types of cells, such as neurons, lymphocytes, and melanoma cells (Ni et al., 2013; Fiala et al., 2015; Lopez-Menendez et al., 2019). In this study, we confirmed that KIDINS220/ARMS inhibits adipocyte maturation and lipid accumulation through sustaining the phosphorylation level of ERK, while a highly selective ERK inhibitor-Ravoxertinib, could fully “rescue” the differentiation potential of preadipocytes. On the contrary, prompted adipocyte maturation driven by KIDINS220 knockdown was abolished by stimulating the ERK signaling with Honokiol. The truncated KIDINS220/ARMS in our case and other previously reported cases do not have the C-terminal region, which harbors several important interacting domains (Iglesias et al., 2000; Zhao et al., 2019), which may disrupt the interaction of KIDINS220/ARMS with ERK, further suppressing the ERK signaling to promote adipocyte differentiation. The data we present here could partly explain the pathogenic obesity in SINO syndrome; nevertheless, the detailed mechanisms need to be further examined. Taken together, this is the first report about the vertical heredity of severe dominant pathogenic mutation in *KIDINS220/ARMS*. KIDINS220/ARMS could function as a negative regulator in adipocyte differentiation and maturation *via* sustained ERK signaling, thus the truncated mutation of KIDINS220/ARMS could prompt adipocyte maturation and leads to obesity. More importantly, we are happy to notice that the younger son improved a lot in all aspects of the SINO syndrome from 2 to 5 years old, which suggests that the neurological symptoms of SINO syndrome could obtain self-improvement during early childhood. It also reminds us that genetic counseling within 3 years after birth, perhaps combined with rehabilitation training could greatly improve the living quality of SINO patients.

## Data Availability Statement

The datasets presented in this study can be found in online repositories. The names of the repository/repositories and accession number(s) can be found below: https://www.biosino.org/node/search, OEP001230; https://www.biosino.org/node/search, OEP001231.

## Ethics Statement

The studies involving human participants were reviewed and approved by the Medical Ethics Committee of Qilu Children’s Hospital of Shandong University. Written informed consent to participate in this study was provided by the participants’ legal guardian/next of kin. Written informed consent was obtained from the individual(s), and minor(s)’ legal guardian/next of kin, for the publication of any potentially identifiable images or data included in this article.

## Author Contributions

YY, SZ, and ZG supervised the project. KZ and YY wrote the manuscript. KZ, YLi, and YLv were involved in clinical diagnosis and whole exome sequencing and bioinformatics analysis. KZ and YLv performed the karyotype analysis and chromosome microarray analysis. YLi and YLv were participated in case follow-up. KZ, WS, and DH carried out the adipocyte differentiation assay. All authors were involved in the conception, experiment design, data analysis, and approved the final manuscript.

## Conflict of Interest

The authors declare that the research was conducted in the absence of any commercial or financial relationships that could be construed as a potential conflict of interest.

## Supplementary Material

Supplementary Figure 1 The amino acid Glu1312 residue of KIDINS220/ARMS were highly conserved from Xenopus to Human. Sequence alignment of the amino acid residues 1283–1338 of human KIDINS220/ARMS with amino acid residues from other nine species. Gaps are indicated by *dashes*, conserved Glu1312 were *boxed*.

Supplementary Figure 2 Schematic diagram of KIDINS220/ARMS domains and landscape of pathogenic variants. The putative KIDINS220/ARMS contains 11 ankyrin repeats (yellow), four transmembrane domains (dark blue), a PRD (light turquoise), a SAM (red), KLC-KIM (pink), and a PDZ-L (dark red). The 17 pathogenic variants of KIDINS220/ARMS reported are shown over the diagram. The variants reported to cause SINO syndrome are shown in light blue, and the variant identified in this study is in red.

Supplementary Table 1 Sequences of primers of siRNA and Q-PCR used in this study are listed.

Supplementary Table 2 Caloric intake and physical activity were calculated in this study.

Supplementary Table 3 The clinical features of the17 KIDINS220/ARMS mutations causing diseases.

## References

[B1] Ainsworth B. E. Haskell W. L. Whitt M. C. Irwin M. L. Swartz A. M. Strath S. J. (2000). Compendium of physical activities: an update of activity codes and MET intensities. *Med. Sci. Sports Exerc.* 32 S498–S504. 10.1097/00005768-200009001-00009 10993420

[B2] Arevalo J. C. Pereira D. B. Yano H. Teng K. K. Chao M. V. (2006). Identification of a switch in neurotrophin signaling by selective tyrosine phosphorylation. *J. Biol. Chem.* 281 1001–1007. 10.1074/jbc.M504163200 16284401

[B3] Bost F. Aouadi M. Caron L. Binetruy B. (2005a). The role of MAPKs in adipocyte differentiation and obesity. *Biochimie* 87 51–56. 10.1016/j.biochi.2004.10.018 15733737

[B4] Bost F. Aouadi M. Caron L. Even P. Belmonte N. Prot M. (2005b). The extracellular signal-regulated kinase isoform ERK1 is specifically required for in vitro and in vivo adipogenesis. *Diabetes* 54 402–411. 10.2337/diabetes.54.2.402 15677498

[B5] Bracale A. Cesca F. Neubrand V. E. Newsome T. P. Way M. Schiavo G. (2007). Kidins220/ARMS is transported by a kinesin-1-based mechanism likely to be involved in neuronal differentiation. *Mol. Biol. Cell* 18 142–152. 10.1091/mbc.e06-05-0453 17079733 PMC1751333

[B6] Caccialanza R. Nicholls D. Cena H. Maccarini L. Rezzani C. Antonioli L. (2004). Validation of the Dutch eating behaviour questionnaire parent version (DEBQ-P) in the Italian population: a screening tool to detect differences in eating behaviour among obese, overweight and normal-weight preadolescents. *Eur. J. Clin. Nutr.* 58 1217–1222. 10.1038/sj.ejcn.1601949 15054434

[B7] Cesca F. Yabe A. Spencer-Dene B. Scholz-Starke J. Medrihan L. Maden C. H. (2012). Kidins220/ARMS mediates the integration of the neurotrophin and VEGF pathways in the vascular and nervous systems. *Cell Death Differ.* 19 194–208. 10.1038/cdd.2011.141 22048155 PMC3263493

[B8] Deswal S. Meyer A. Fiala G. J. Eisenhardt A. E. Schmitt L. C. Salek M. (2013). Kidins220/ARMS associates with B-Raf and the TCR, promoting sustained Erk signaling in T cells. *J. Immunol.* 190 1927–1935. 10.4049/jimmunol.1200653 23359496

[B9] Duffy A. M. Schaner M. J. Wu S. H. Staniszewski A. Kumar A. Arevalo J. C. (2011). A selective role for ARMS/Kidins220 scaffold protein in spatial memory and trophic support of entorhinal and frontal cortical neurons. *Exp. Neurol.* 229 409–420. 10.1016/j.expneurol.2011.03.008 21419124 PMC3100364

[B10] Ferguson B. S. Nam H. Stephens J. M. Morrison R. F. (2016). Mitogen-dependent regulation of DUSP1 governs ERK and p38 signaling during Early 3T3-L1 adipocyte differentiation. *J. Cell. Physiol.* 231 1562–1574. 10.1002/jcp.25248 26566083 PMC7577398

[B11] Fiala G. J. Janowska I. Prutek F. Hobeika E. Satapathy A. Sprenger A. (2015). Kidins220/ARMS binds to the B cell antigen receptor and regulates B cell development and activation. *J. Exp. Med.* 212 1693–1708. 10.1084/jem.20141271 26324445 PMC4577850

[B12] Hausman D. B. DiGirolamo M. Bartness T. J. Hausman G. J. Martin R. J. (2001). The biology of white adipocyte proliferation. *Obes. Rev.* 2 239–254. 10.1046/j.1467-789x.2001.00042.x 12119995

[B13] Iglesias T. Cabrera-Poch N. Mitchell M. P. Naven T. J. Rozengurt E. Schiavo G. (2000). Identification and cloning of Kidins220, a novel neuronal substrate of protein kinase D. *J. Biol. Chem.* 275 40048–40056. 10.1074/jbc.M005261200 10998417

[B14] Jaudon F. Chiacchiaretta M. Albini M. Ferroni S. Benfenati F. Cesca F. (2020). Kidins220/ARMS controls astrocyte calcium signaling and neuron-astrocyte communication. *Cell Death Differ.* 27 1505–1519. 10.1038/s41418-019-0431-5 31624352 PMC7206051

[B15] Josifova D. J. Monroe G. R. Tessadori F. de Graaff E. van der Zwaag B. Mehta S. G. (2016). Heterozygous KIDINS220/ARMS nonsense variants cause spastic paraplegia, intellectual disability, nystagmus, and obesity. *Hum. Mol. Genet.* 25 2158–2167. 10.1093/hmg/ddw082 27005418

[B16] Kleinendorst L. Massink M. P. G. Cooiman M. I. Savas M. van der Baan-Slootweg O. H. Roelants R. J. (2018). Genetic obesity: next-generation sequencing results of 1230 patients with obesity. *J. Med. Genet.* 55 578–586. 10.1136/jmedgenet-2018-105315 29970488

[B17] Kong H. Boulter J. Weber J. L. Lai C. Chao M. V. (2001). An evolutionarily conserved transmembrane protein that is a novel downstream target of neurotrophin and ephrin receptors. *J. Neurosci.* 21 176–185. 11150334 10.1523/JNEUROSCI.21-01-00176.2001 PMC6762419

[B18] Lone J. Yun J. W. (2017). Honokiol exerts dual effects on browning and apoptosis of adipocytes. *Pharmacol Rep* 69 1357–1365. 10.1016/j.pharep.2017.06.004 29136581

[B19] Lopez-Menendez C. Gamir-Morralla A. Jurado-Arjona J. Higuero A. M. Campanero M. R. Ferrer I. (2013). Kidins220 accumulates with tau in human Alzheimer’s disease and related models: modulation of its calpain-processing by GSK3beta/PP1 imbalance. *Hum. Mol. Genet.* 22 466–482. 10.1093/hmg/dds446 23118350

[B20] Lopez-Menendez C. Gascon S. Sobrado M. Vidaurre O. G. Higuero A. M. Rodriguez-Pena A. (2009). Kidins220/ARMS downregulation by excitotoxic activation of NMDARs reveals its involvement in neuronal survival and death pathways. *J. Cell. Sci.* 122 3554–3565. 10.1242/jcs.056473 19759287

[B21] Lopez-Menendez C. Simon-Garcia A. Gamir-Morralla A. Pose-Utrilla J. Lujan R. Mochizuki N. (2019). Excitotoxic targeting of Kidins220 to the Golgi apparatus precedes calpain cleavage of Rap1-activation complexes. *Cell Death Dis.* 10:535. 10.1038/s41419-019-1766-z 31296845 PMC6624258

[B22] Mero I. L. Mork H. H. Sheng Y. Blomhoff A. Opheim G. L. Erichsen A. (2017). Homozygous KIDINS220 loss-of-function variants in fetuses with cerebral ventriculomegaly and limb contractures. *Hum. Mol. Genet.* 26 3792–3796. 10.1093/hmg/ddx263 28934391

[B23] Ni X. Li X. Fang X. Li N. Cui W. Zhang B. (2010). NGF/TrkA-mediated Kidins220/ARMS signaling activated in the allergic airway challenge in mice. *Ann. Allergy Asthma Immunol.* 105 299–306. 10.1016/j.anai.2010.08.006 20934630

[B24] Ni X. Li X. Tao S. Xu M. Ma H. Wang X. (2013). Blockade of ankyrin repeat-rich membrane spanning protein modulates extracellular signal-regulated kinase expression and inhibits allergic inflammation in ovalbumin-sensitized mice. *Biomed. Rep.* 1 674–678. 10.3892/br.2013.120 24649008 PMC3917733

[B25] Oh J. H. Karadeniz F. Lee J. I. Seo Y. Kong C. S. (2019). Artemisia princeps inhibits adipogenic differentiation of 3T3-L1 pre-adipocytes via downregulation of PPARgamma and MAPK pathways. *Prev. Nutr. Food Sci.* 24 299–307. 10.3746/pnf.2019.24.3.299 31608255 PMC6779088

[B26] Raza M. Z. Allegrini S. Dumontet C. Jordheim L. P. (2018). Functions of the multi-interacting protein KIDINS220/ARMS in cancer and other pathologies. *Genes Chromosomes Cancer* 57 114–122. 10.1002/gcc.22514 29181864

[B27] Richards S. Aziz N. Bale S. Bick D. Das S. Gastier-Foster J. (2015). Standards and guidelines for the interpretation of sequence variants: a joint consensus recommendation of the American college of medical genetics and genomics and the association for molecular pathology. *Genet. Med.* 17 405–424. 10.1038/gim.2015.30 25741868 PMC4544753

[B28] Rockett H. R. Breitenbach M. Frazier A. L. Witschi J. Wolf A. M. Field A. E. (1997). Validation of a youth/adolescent food frequency questionnaire. *Prev. Med.* 26 808–816. 10.1006/pmed.1997.0200 9388792

[B29] Simons A. Shaffer L. G. Hastings R. J. (2013). Cytogenetic nomenclature: changes in the ISCN 2013 compared to the 2009 edition. *Cytogenet. Genome Res.* 141, 1–6. 10.1159/000353118 23817294

[B30] Sulahian R. Kwon J. J. Walsh K. H. Pailler E. Bosse T. L. Thaker M. (2019). Synthetic lethal interaction of SHOC2 depletion with MEK inhibition in RAS-driven cancers. *Cell Rep.* 29:e118. 10.1016/j.celrep.2019.08.090 31577942 PMC6918830

[B31] Vasheghani-Farahani A. Tahmasbi M. Asheri H. Ashraf H. Nedjat S. Kordi R. (2011). The Persian, last 7-day, long form of the international physical activity questionnaire: translation and validation study. *Asian J. Sports Med.* 2 106–116. 10.5812/asjsm.34781 22375226 PMC3289200

[B32] Yamaki K. (2005). Body weight status among adults with intellectual disability in the community. *Ment. Retard.* 43 1–10. 10.1352/0047-6765200543<1:BWSAAW<2.0.CO;2 15628929

[B33] Yang L. Zhang W. Peng J. Yin F. (2018). Heterozygous KIDINS220 mutation leads to spastic paraplegia and obesity in an Asian girl. *Eur. J. Neurol.* 25 e53–e54. 10.1111/ene.13600 29667355

[B34] Zampieri N. Chao M. V. (2006). Mechanisms of neurotrophin receptor signalling. *Biochem. Soc. Trans.* 34 607–611. 10.1042/BST0340607 16856873

[B35] Zhao M. Chen Y. J. Wang M. W. Lin X. H. Dong E. L. Chen W. J. (2019). Genetic and clinical profile of chinese patients with autosomal dominant spastic paraplegia. *Mol. Diagn. Ther.* 23 781–789. 10.1007/s40291-019-00426-w 31630374

