# Supplementary material for: SINO Syndrome Causative KIDINS220/ARMS Gene Regulates Adipocyte Differentiation
Source: Front Cell Dev Biol. 2021 Mar 4;9:619475. doi: 10.3389/fcell.2021.619475 (PMC7982959; doi:10.3389/fcell.2021.619475)
Supplement: Supplementary Figure 1 — The amino acid Glu1312 residue of KIDINS220/ARMS were highly conserved from Xenopus to Human. Sequence alignment of the amino acid residues 1283–1338 of human KIDINS220/ARMS with amino acid residues from other nine species. Gaps are indicated by dashes, conserved Glu1312 were boxed. [file Table_1.pdf]

SUPPLEMENTS:

Table S1. Sequences of primers of siRNA and Q-PCR used in this study are listed.

**Quantitative RT-PCR Primers**	
Kidins220 forward-1:	5'- GTGCTCTTCCACGTCCTTCA
Kidins220 reverse-1:	5'- GGGCAAAGAATGGCCTGTTG
Kidins220 forward-2:	5'- CAACAGGCCATTCTTTGCCC
Kidins220 reverse-2:	5'- GTAATACCACTGGGCCTGGG
Rplp0 forward:	5'-TTCATTGTGGGAGCAGAC
Rplp0 reverse:	5'- CAGCAGTTTCTCCAGAGC
***siRNA* sequence**	
Kidins220 siRNA-1:	5'-GCATCAAAGGAGGGACATA
Kidins220 siRNA-2:	5'-GCAAGTGATCCACATATTA

Table S2. Caloric intake and physical activity were calculated in this study

		**Mother**	**First son**	**Second son**
Age(years)		39	17	5
caloric intake(kcal/d) (REF)		2117±27 (1600-2400)	2470±103 (1613-2779)	1510±132 (904.5-1865.9)
**IPAQ**				
Total PA (MET-min week-1)		8082.6±3198.4	5996.5±1509.7	2110.1±739.9
Intensities	Vigorous PA	1086.5±1256.4	290.8±478.9	0
	Moderate	5570.3±1665.7	4081.6±1003.5	676.1±587.8
	Walking	1425.8±606.3	1624.2±337.7	1434±434

MET-min week^{-1}: metabolic equivalent scores (Ainsworth et al., 2000)

PA: physical activity

IPAQ: International Physical Activity Questionnaire

REF: the normal reference values

Table S3: The clinical features of the17 KIDINS220/ARMS mutations causing diseases

Mutation	Spastic paraplegia	Intellectual disability	Nystagmus	Obesity	Brachycephaly	Ventricular enlargement	reference
c.1369C>T (p.Q457*)	-	+	+	-	-	-	Decipher database
c.3102_3103ins7bp (p.R1035Qfs*54)	+	+	-	-	-	-	Decipher database
c.4448C>G (p.S1483*)	+	-	-	-	-	-	(Zhao et al., 2019)
c.3394_3403del (p.Q1132Sfs*30)	+	-	-	-	-	+	(Mero et al., 2017)
c.4050G>A (p.W1350*)	+	+	+	+	+	+	(Josifova et al., 2016)
c.4096C>T (p.Q1366*)	+	+	+	+	+	+	(Josifova et al., 2016)
c.4380_4390del (p.S1463fs*15)	+	+	-	+	-	-	(Mero et al., 2017)
c.4520_4521ins (p.L1507Ffs*4)	+	+	+	+	+	+	(Josifova et al., 2016)
c.207+3A>G	-	-	-	+	-	-	(Kleinendorst et al., 2018)
c.603+3_c.603+7delAAGTA	-	-	-	+	-	-	(Kleinendorst et al., 2018)
c.1117C>T (p.H373Y)	-	-	-	+	-	-	(Kleinendorst et al., 2018)
c.2635A>G (p.R879G)	-	-	-	+	-	-	(Kleinendorst et al., 2018)
c.3139T>C (p.F1047L)	-	-	-	+	-	-	(Kleinendorst et al., 2018)
c.5002C>T (p. P1668S)	-	-	-	+	-	-	(Kleinendorst et al., 2018)
c.5221A>T (p. S1741C)	-	-	-	+	-	-	(Kleinendorst et al., 2018)
c.5242G>T (p.D1748Y)	-	-	-	+	-	-	(Kleinendorst et al., 2018)
**c.3934G>T (p.E1312*)**	+	+	+/-	-	+	-	**our cohort**

SUPPLEMENTAL FIGURES:

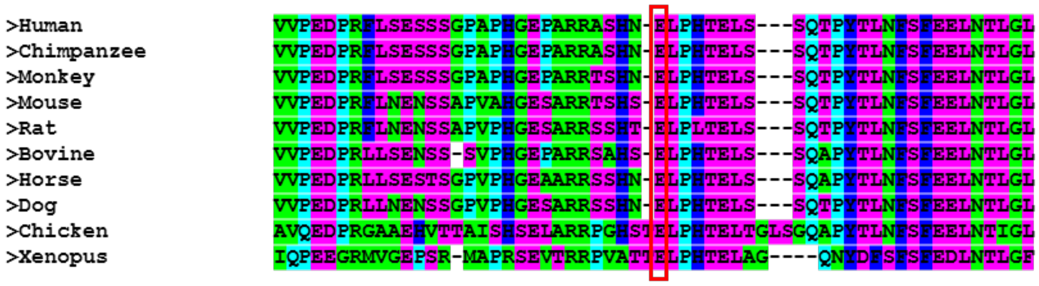

Fig. S1

Figure S1. The amino acid Glu1312 residue of KIDINS220/ARMS were highly conserved from Xenopus to Human. Sequence alignment of the amino acid residues 1283-1338 of human KIDINS220/ARMS with amino acid residues from other 9 species. Gaps are indicated by *dashes*, conserved Glu1312 were *boxed*.

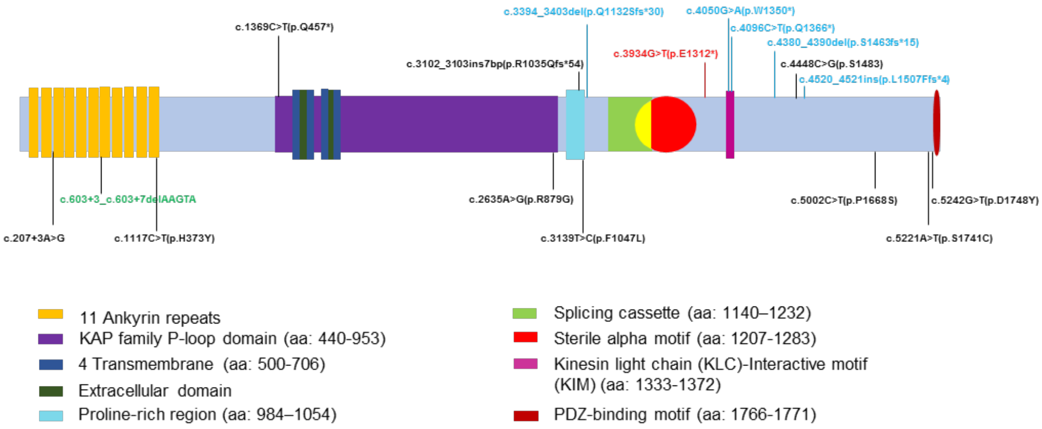

Fig. S2

Figure S2. Schematic diagram of KIDINS220/ARMS domains and landscape of pathogenic variants. The putative KIDINS220/ARMS contains 11 ankyrin repeats (yellow), 4 transmembrane domains (dark blue), a proline-rich domain (PRD, light turquoise), a sterile alpha motif (SAM, red), Kinesin Light Chain (KLC) - Interactive Motif (KIM, pink), and a PDZ ligand (PDZ-L, dark red). The 17 pathogenic variants of KIDINS220/ARMS reported are shown over the diagram. The variants reported to cause SINO syndrome are shown in light blue, and the variant identified in this study is in red.
